# (Auto)Antibody Responses Shape Memory NK Cell Pool Size and Composition

**DOI:** 10.3390/biomedicines10030625

**Published:** 2022-03-08

**Authors:** Cristina Capuano, Chiara Pighi, Simone Battella, Fabio Pulcinelli, Cristina Santoro, Antonietta Ferretti, Ombretta Turriziani, Davide De Federicis, Cinzia Fionda, Giuseppe Sciumè, Ricciarda Galandrini, Gabriella Palmieri

**Affiliations:** 1 Department of Experimental Medicine, Sapienza University of Rome, 00185 Roma, Italy; cristina.capuano@uniroma1.it (C.C.); chiara.pighi@opbg.net (C.P.); simone.battella@reithera.com (S.B.); fabio.pulcinelli@uniroma1.it (F.P.); davide.defedericis@uniroma1.it (D.D.F.); 2 Hematology Division, Policlinico Umberto I, 00185 Rome, Italy; santoro@bce.uniroma1.it (C.S.); ferretti@bce.uniroma1.it (A.F.); 3 Department of Molecular Medicine, Sapienza University of Rome, 00185 Roma, Italy; ombretta.turriziani@uniroma1.it (O.T.); cinzia.fionda@uniroma1.it (C.F.); giuseppe.sciume@uniroma1.it (G.S.)

**Keywords:** memory natural killer (NK) cells, CD16, auto-antibodies, HCMV, immune thrombocytopenia (ITP)

## Abstract

In vivo establishment and long-term persistence of a heterogeneous memory or an adaptive NK cell pool represents a functional adaptation to human cytomegalovirus (HCMV) infection in humans. Memory NK cells are commonly identified by lack of the FcεRIγ signalling chain, variably associated to the preferential but not completely overlapping expression of the HLA-E receptor NKG2C and CD57 maturation marker. Although characterized by selective hyperresponsiveness to IgG stimulation, the impact of the CD16/antibody interaction in regulating the establishment/maintenance and size, and in determining the relative abundance of this population, is still under investigation. Memory NK cell subset ex vivo profile and in vitro responsiveness to CD16 stimulation was evaluated in HCMV^+^ healthy donors and in patients affected by immune thrombocytopenia (ITP), an antibody-mediated autoimmune disease. We identified the FcεRIγ^−^ NKG2C^+^CD57^+^ memory NK cell subset, whose abundance is uniquely associated with anti-HCMV antibody levels in healthy seropositive donors, and which is significantly expanded in ITP patients. This fully mature memory subset robustly and selectively expands in vitro in response to mAb-opsonized targets or ITP-derived platelets and displays superior CD16-dependent IFNγ production. Our work identifies opsonizing antibodies as a host-dependent factor that shapes HCMV-driven memory NK cell compartment. We first demonstrate that chronic exposure to auto-antibodies contributes to the establishment/expansion of a highly specialized and unique memory NK cell subset with distinct CD16-dependent functional capabilities. We also identify the specific contribution of the lack of FcεRIγ chain in conferring to NKG2C^+^CD57^+^ memory cells a higher responsivity to CD16 engagement.

## 1. Introduction

The spectrum of NK cell heterogeneity varies among individuals, reflecting in part their adaptation to pathogens. A role for infection in driving the functional adaptation of human NK cells is particularly well documented in the case of human cytomegalovirus (HCMV), a herpesvirus that infects most of the world’s population [1,2]. A distinct but heterogeneous population of mature NK cells that exhibits adaptive immune features, which include the long-term persistence in vivo, a distinct epigenetic and metabolic profile resembling that of memory CD8^+^ T cells, and a peculiar equipment of intracellular enzymes and signalling components, has been described in a fraction of healthy HCMV^+^ individuals [3,4,5,6]. Such “memory” or “adaptive” NK cells are marked by a functional hyperresponsivity to CD16 (also named FcγRIIIA), stimulation [5,6,7,8]. Their enhanced capability to produce IFNγ, TNFα, and chemokines upon CD16 stimulation is coupled to low responsiveness to NKp46 and NKp30 NCR engagement, as well as to IL-12/IL-18 inflammatory cytokines, as compared to conventional counterparts [7,9,10,11].

The memory NK cell pool, whose size greatly varies among HCMV^+^ individuals [12,13], has been identified within mature CD56^dim^CD16^+^ NK cells through the expression of variable and not completely overlapping combinations of markers; the epigenetically controlled downmodulation of an FcεRIγ signalling molecule on one side, and the preferential expression of NKG2C activating receptor and of CD57 maturation marker on the other, are most commonly employed [2,5,6,14,15,16,17].

The immunoreceptor tyrosine-based activating motif (ITAM)-containing FcεRIγ chain physically associates with the CD16/FcγRIIIA receptor, as a homodimer or heterodimer with the TCRζ chain [18], and to NKp46 and NKp30 natural cytotoxicity receptors (NCR)s [19]. CD16 is a prototypical activating receptor on mature NK cells, as its aggregation by IgG-opsonized target cells unleashes NK cell effector capabilities, i.e., the production of cytokines and chemokines, and antibody-dependent cytotoxicity (ADCC) [19,20]. CD16-dependent signals impact NK cell behaviour globally, as they can also modulate survival, proliferation and apoptosis in selected contexts [21,22,23]. Several reports have demonstrated that FcεRIγ^−^ memory NK cells expand in vitro following exposure to virus-infected cells in the presence of antiviral antibodies, or upon co-culture with rituximab-opsonized B lymphoma cells [5,6,24], thus underscoring the role of CD16-initiated signals in inducing memory NK cell proliferation. A correlation between anti-HCMV neutralizing antibody levels and the frequency of NKG2C^+^CD57^+^ or FcεRIγ^−^ CD57^+^ NK cells has been previously noted in bone marrow transplant (BMT) recipients upon HCMV reactivation [25]. In vitro FcεRIγ gene targeting has been shown to enhance CD16 responsiveness of conventional NK cells, thus underscoring the role of FcεRIγ downmodulation in explaining the higher sensitivity of memory NK cells to opsonizing antibodies [26]. 

Conversely, a direct role for the NKG2C receptor in driving memory NK cell proliferation is supported by in vivo observations in patients experiencing primary HCMV infection or re-activation [14,15,27,28]. CD94/NKG2C recognition of the non-classical MHC class I HLA-E molecule activates NK cell effector functions, due to the association with the ITAM-containing DAP12 adaptor molecule [19,20,29]. In vitro studies have demonstrated that NKG2C^+^ NK cells undergo clonal-like expansion upon recognition of certain HCMV UL-40 peptides presented by HLA-E [30]. This mechanism, which is highly sensitive to strain-dependent UL-40 variations, may contribute to the variability of memory NKG2C^+^ NK cell frequency in HCMV-infected people. 

A complex epigenetic and transcriptional remodelling contributes to the memory NK cell distinct phenotypic and functional profile. Single-cell transcriptome analysis confirmed the downmodulation of the FcεRIγ chain, Syk tyrosine kinase and EAT-2 adaptor, all B and myeloid cell-related signalling proteins, along with the decreased expression of PLZF transcription factor, among others [3,5,6,31]. These features do not uniformly coexist in the same cell, suggesting that the memory NK cell pool encompasses a spectrum of epigenetically unique subsets, endowed with distinct functional capabilities and regulation, and that both host- and virus-dependent factors contribute to the establishment and to interindividual size variability of this specialized population.

Qualitative and/or quantitative perturbation of the NK cell compartment characterizes a wide range of autoimmune diseases, but the possible mechanisms for their involvement have not been elucidated yet [32,33]. In this regard, memory NK cells, due to their enhanced capability to respond to antibody-opsonized cells and to their skewed immunoregulatory profile, may represent an interesting candidate for playing either a protective and/or a pathogenetic role in the regulation of autoimmune diseases [34,35].

Here, we have characterized the in vivo and in vitro dynamics of HCMV-driven memory NK cell subsets, as defined by NKG2C and CD57 expression; in particular, we investigated the impact of the absence of FcεRIγ chain in shaping memory NK cell pool composition in vivo, and its responsiveness to CD16 engagement, evaluated either as proliferation or IFNγ production. Memory NK cell subsets were analysed in HCMV^+^ healthy subjects, and in patients affected by immune thrombocytopenia (ITP), an autoimmune condition characterized by platelet- and megakaryocyte-opsonizing antibodies [36]; importantly, we directly demonstrated that chronic exposure to ITP platelets drives the in vitro expansion of memory NK cell subsets. Overall, we evidenced that the absence of the FcεRIγ chain confers enhanced CD16 responsiveness to mature NK cells and, along with the co-expression of NKG2C and CD57, defined a specialized memory NK subset with the highest sensitivity to opsonizing (auto)antibodies.

## 2. Materials and Methods

### 2.1. Healthy Donors and ITP Patients

Healthy donors (*n* = 229; 154 HCMV^+^ and 75 HCMV^−^) were recruited at Blood Transfusion Centre, Policlinico Umberto I, in an anonymized form. ITP patients (*n* = 26, all HCMV^+^) were referred to the Haematology Division, Policlinico Umberto I, where definite diagnosis was given according to international guidelines [37]. All patients and controls gave written informed consent to the study. All procedures involving human participants were in accordance with the regulations of health information protection policies, and the Declaration of Helsinki and its later amendments. The study was approved by the Ethics Committee of Sapienza University of Rome (approval number 639/16 RIF/CE 4179). 

### 2.2. Peripheral Blood Mononuclear Cell (PBMC) Isolation

Peripheral blood mononuclear cell (PBMC) populations were freshly isolated from heparinized blood samples by lymphoprep (Ficoll-Hypaque, Cedarlane, Burlington, ON, Canada) density gradient centrifugation. After washing in phosphate buffered saline (PBS), cell samples were used for in vitro stimulation, and for ex vivo and in vitro immunostaining and cytofluorimetric assays. 

### 2.3. Cell Lines

Lymphoblastoid CD20^+^ Raji cell line was provided by Dr. F.D. Batista (Ragon Institute of MGH, MIT and Harvard, Cambridge, MA, USA). Cells were checked for morphology, growth and immunophenotypic characteristics, according to provider’s recommendations (last testing May 2021), kept in culture for less than two consecutive months in 10% Foetal Calf Serum (FCS)- and 1% L-glutamine (both from Euroclone, Milan, Italy) containing RPMI 1640, and routinely tested for mycoplasma contamination by EZ-PCR Mycoplasma test kit (cat.#: 20-700-20, Biological Industries, Haemek, Israel). 

### 2.4. Phenotypic Characterization of Memory NK Cell Subsets

Freshly isolated and in vitro cultured cell populations were subjected to immunostaining with previously defined saturating concentrations of the following fluorochrome-conjugated mAbs: anti-CD3 PerCP-Vio700 (Clone: BW264/56, cat.#:130-113-132), anti-CD56 APC-Vio770 (Clone: REA 196, cat.#: 130-114-548), anti-CD16 PE-Vio770 (Clone: REA 423, cat.#: 130-113-394), anti-NKG2C-PE (Clone: REA 205, cat.#: 130-119-776), anti-CD57 APC (Clone: REA 769, cat.#: 130-111-811) or anti-CD57 PE-Vio770 (Clone REA 769 cat.#: 130-111-812), all from Miltenyi Biotec Italy, for 30 min at 4 °C. After staining, samples were washed with 2% foetal calf serum (FCS)- and 2mM EthyleneDiamineTetraAcetic acid disodium salt (EDTA)-containing PBS (Euroclone) (used for all the washing steps) and fixed with 2% paraformaldehyde (PFA) (Merck, Germany) for 20 min at room temperature (RT). Fixed samples were washed, permeabilized for 30 min at RT with 0.05% Triton-X 100 containing washing solution and incubated for 30 min at 4 °C with anti-FcεRIγ subunit FITC-conjugated polyclonal antibody (cat.#: FCABS400F, Merck). Staining with anti-Bcl-2 APC (Clone: REA 872 cat.#: 130-114-232, Miltenyi Biotec) was performed after fixing and permeabilizing with Fixation/Permeabilization buffer kit (cat.#: 00-5523-00, eBioscience, Thermo Fisher, Waltham, MA, USA), according to manufacturer’s instructions.

All samples (at least 700,000 total events/sample) were acquired with a FACSCanto II (BD Biosciences, Franklin Lakes, NJ, USA). Instrument calibration was performed with BD Cytometer Setup and Tracking Beads, BD Biosciences, cat. 641319; signal compensation process was performed with compensation beads (MACS Comp Bead Kit anti-REA 130-104-693 Miltenyi and BD CompBeads, Anti-Mouse Ig, k/Negative Control Compensation Particle Set, cat. 552843, BD Biosciences) and FACSDiva software algorithm.

Cytofluorimetric data were analysed with FlowJo vX.0.7 (Becton-Dickinson Biosciences, San Jose, CA, USA) software. Memory NK cell subsets were identified by antibody combinations within the lymphocyte region, determined by physical parameters (FSC and SSC).

### 2.5. NK Cell In Vitro Cultures

PBMC from 28 HCMV^+^ healthy donors were seeded in round-bottomed 96-well plates (50,000 cells/well) and cultured for 9 days in RPMI 1640 medium supplemented with 10% FCS, 1% L-glutamine, 1% Penicillin–Streptomycin (complete medium) and 100 IU/mL of human recombinant IL-2 (cat.#: AF-200-02, Peprotech, London, UK); after two days, irradiated (3000 rad/30 Gy) Raji cells, opsonized or not with rituximab chimeric mAb at saturating dose (kindly provided by Dr. Christian Klein, Roche Innovation Centre Zurich, Schlieren, Switzerland) for 20 min at RT, were added to the cultures (25,000 cells/well) [24]. 

Healthy donor or ITP patient whole blood samples were collected in sodium citrate-containing tubes and centrifuged at 200× *g* for 15 min at 22 °C to obtain Platelet-Rich Plasma (PRP), as previously described [38]. PRP was supplemented with 10% Acid-Citrate Dextrose (ACD) buffer (PBS containing 2.5% dibasic sodium citrate, 1.5% citric acid and 2% glucose), and centrifuged at 800× *g* for 10 min at 22 °C. Platelets were re-suspended in complete medium (100 × 10^6^/100 μL), seeded in round-bottomed 96-well plates (2.5 × 10^6^ platelets/well), and cultured for 12 days with allogeneic healthy donor-derived PBMC (50,000 cells/well), in the presence of IL-2 (12 independent cultures). After two days, irradiated Raji cells were added to the cultures. 

### 2.6. Evaluation of IFNγ^−^Producing Cells

Freshly isolated PBMC were allowed to interact at a 2:1 Effector:Target ratio with Raji cell line, opsonized or not with a minimum saturating dose of rituximab (1 μg/L × 10^6^ cells), for 6 h at 37 °C in the presence of 50 μM Monensin (Golgi-stop; cat.#: M5273, Merck), as previously described [39]. After the first hour, 10 μg/mL Brefeldin A (cat.#: B7651, Merck) was added. At the end of stimulation, cells were washed with 2% FCS- and 2 mM EDTA-containing PBS, and then stained for surface antigens. Anti-IFNγ APC (clone: B27, cat.#: 554702, BD Biosciences) was added after fixation and permeabilization, as above.

### 2.7. HCMV Serostatus Analysis

Plasma anti-HCMV IgG levels were determined by CMV IgG Immulite 2000 System (Siemens Healthineers, Milan, Italy). HCMV seropositivity corresponded to >1 arbitrary units (au), according to manufacturer’s instructions, and as previously reported [24]. 

### 2.8. Statistical Analysis

Differences between groups were analysed with non-parametric (Mann–Whitney U, Wilcoxon signed rank, Friedman) tests, as appropriate; Spearman’s test was used to analyse correlations between variables; statistical analysis was performed with SPSS v25 and v27 (IBM, Armonk, NY, USA) and Prism v6.0 (GraphPad, San Diego, CA, USA) software packages. Differences were considered to be statistically significant when *p* value was <0.05 (two-sided).

## 3. Results

### 3.1. The Asset of Memory NK Cell Subsets In Vivo Impact of Anti-HCMV Antibody Levels

**Figure 1:**
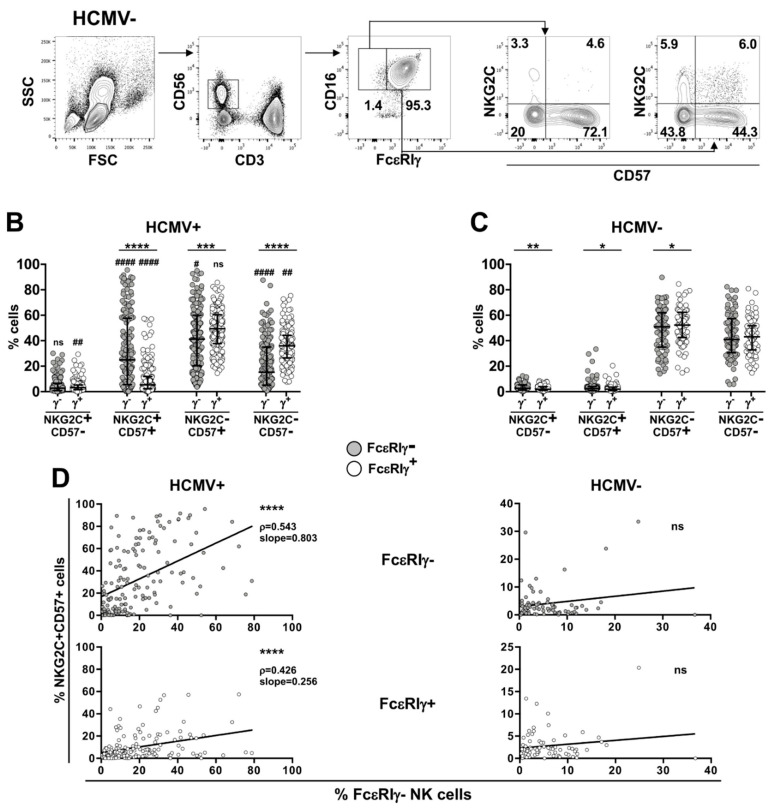
FcεRIγ^−^NKG2C^+^CD57^+^ cells markedly expand in HCMV-seropositive individuals and tightly correlate with individual FcεRIγ^−^ memory pool size. (**A**) Gating strategy for the identification of peripheral blood FcεRIγ^−^ and FcεRIγ^+^ CD16^+^CD56^dim^ NK cell subsets. Numbers represent percentage of gated cells. (**B**,**C**) Stratification of CD16^+^CD56^dim^ NK cells according to NKG2C and CD57 expression, within FcεRIγ^−^ (γ^−^, grey) and FcεRIγ^+^ (γ^+^, empty) cell pools, of 154 HCMV-seropositive (HCMV^+^) (**B**) and 75 HCMV-seronegative (HCMV^−^) (**C**) healthy individuals. Bars represent median with interquartile range. *p* values of pairwise comparisons were calculated with Wilcoxon (FcεRIγ^−^ vs. FcεRIγ^+^) and Mann–Whitney (HCMV^+^ vs. HCMV^−^) non-parametric tests. (*) symbol indicates statistical comparison with corresponding FcεRIγ^+^ subset (FcεRIγ^−^ vs. FcεRIγ^+^): (*) <0.05, (**) <0.005, (***) < 0.0001, (****) < 0.00001; (#) symbol indicates statistical comparison with corresponding subset of HCMV^−^ donors (HCMV^+^ vs. HCMV^−^): (#) < 0.05, (##) < 0.005, (####) < 1 × 10^−7^. (**D**) Correlation between the percentage of FcεRIγ^−^NKG2C^+^CD57^+^ (grey, upper panels) or FcεRIγ^+^NKG2C^+^CD57^+^ (white, lower panels) subsets with the size of the FcεRIγ^−^ memory NK cell pool in HCMV^+^ (left panels) and HCMV^−^ (right panels) individuals. Spearman’s correlation coefficients (r) and curve slopes are reported when 2-tailed *p* values were significant (<0.05): (****) < 1 × 10^−7^.

Human memory NK cells are identified, within mature CD16^+^CD56^dim^ NK cells, by the lack of FcεRIγ chain, or alternatively, through the preferential expression of NKG2C and/or CD57 surface molecules [2,5,6,14,15]; although the three markers were significatively and co-ordinately more expressed in HCMV-seropositive individuals (Supplementary Figure S1A,B, respectively), the stratification according to NKG2C and CD57 surface markers (Figure 1A) identified NKG2C^+^CD57^+^ cells, within both FcεRIγ^−^ and FcεRIγ^+^ cell pools, as those uniquely more abundant in HCMV^+^ than in HCMV^−^ individuals (Figure 1B,C); notably, FcεRIγ^−^ NKG2C^+^CD57^+^ relative expansion was significatively more marked than that of the homologous FcεRIγ-expressing counterpart (Figure 1B). At variance, FcεRIγ^−^ and FcεRIγ^+^ NK cell pools of HCMV^−^ subjects were comparably and almost completely composed of NKG2C^−^CD57^+^ and NKG2C^−^CD57^+^ cells, in roughly similar proportion (Figure 1C). The relative expansion of FcεRIγ^−^ and, to a lesser extent, of FcεRIγ^+^ NKG2C^+^CD57^+^ cells, largely varied in HCMV^+^ individuals (Figure 1B), thus matching the variable size of the entire FcεRIγ^−^ memory NK cell pool (Supplementary Figure S1). The size of FcεRIγ^−^ NKG2C^+^CD57^+^ (correlation slope = 0.803) and, to a lesser degree, FcεRIγ^+^NKG2C^+^CD57^+^ (correlation slope = 0.256) subsets significatively correlated with the amplitude of the FcεRIγ^−^ memory pool in HCMV^+^ healthy subjects (Figure 1D). 

These observations confirm that HCMV-seropositivity sharply associates with the expansion of NKG2C and CD57 co-expressing cells and highlight that the absence of a FcεRIγ chain confers a significative advantage for their expansion/persistence in vivo. Moreover, they suggest that the size of the HCMV-dependent memory FcεRIγ^−^ pool is mostly regulated by individual factors that heavily impact the expansion of its NKG2C^+^CD57^+^ subset. 

**Figure 2:**
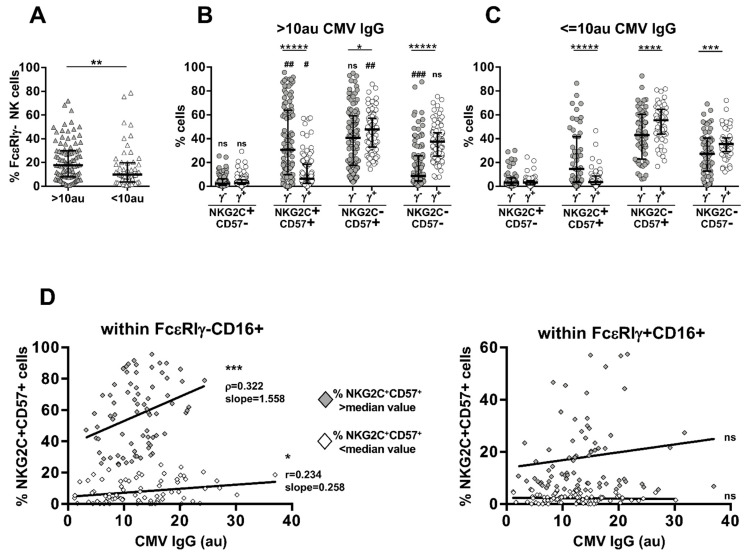
Anti-HCMV IgG levels impact FcεRIγ^−^ memory NK cell pool abundance and correlate with the expansion of FcεRIγ^−^NKG2C^+^CD57^+^ subset in seropositive individuals. (**A**) Percentage of FcεRIγ^−^ memory (within CD16^+^CD56^dim^) NK cells in HCMV^+^ healthy donors stratified for higher (>10 arbitrary units, au, grey triangles) or lower (≤10 au, empty triangles) levels of anti-HCMV antibodies. Relative percentage of FcεRIγ^−^ (γ^−^, grey circles) and FcεRIγ^+^ (γ^+^, empty circles) subsets in individuals with >10 au (**B**) or ≤10 au (**C**) anti-HCMV antibodies. Bars represent median with interquartile range. *p* values of pairwise comparisons were calculated with Wilcoxon and Mann–Whitney (>10 au vs. ≤10 au) non-parametric tests, as appropriate. (*) symbol indicates statistical comparison with corresponding FcεRIγ^+^ subset (FcεRIγ^−^ vs. FcεRIγ^+^): (*) < 0.05, (**) = 0.01, (***) < 0.005, (****) < 0.001, (*****) < 0.00001; (#) symbol indicates statistical comparison with corresponding subset of individuals producing lower levels of anti-HCMV antibodies (>10 au vs. ≤10 au): (#) < 0.05, (##) < 0.01, (###) < 0.0005. (**D**) Correlation between the relative abundance of FcεRIγ^−^ (left panel) or FcεRIγ^+^ (right panel) NKG2C^+^CD57^+^ cells and anti-HCMV antibody concentration. Individuals possessing a larger (higher the median value, grey diamonds) or smaller (lower than median value, empty diamonds) given subset were analysed separately. Spearman’s correlation coefficients (r) and curve slopes are reported when 2-tailed *p* values were significant (<0.05): (*) < 0.05, (***) < 0.005.

The extreme variability of the FcεRIγ^−^CD16^+^ memory NK cell pool size showed no relationship with age or sex in our large cohort of HCMV^+^ healthy subjects (data not shown). In line with previous observations in BMT recipients experiencing CMV reactivation [25], healthy donors producing higher levels of anti-HCMV IgG (>10 arbitrary units) exhibited a significatively larger pool of FcεRIγ^−^ memory NK cells (Figure 2A). The relative expansion of the FcεRIγ^−^NKG2C^+^CD57^+^ subset was markedly higher (median percentages: 30.6 vs. 14.5 in high vs. low anti-HCMV Abs) than that of its FcεRIγ^+^ counterpart (median percentages: 6.5 vs. 3.5 in high vs. low anti-HCMV Abs) (Figure 2B,C). Data reported in Figure 2D further support a stronger dependence of FcεRIγ^−^NKG2C^+^CD57^+^ expansion on HCMV antibody levels, as the relative abundance of this subset, but not of its FcεRIγ^+^ counterpart, significatively correlated with HCMV IgG at individual levels; notably, donors possessing a larger FcεRIγ^−^NKG2C^+^CD57^+^ subset (above 24.95% median value) showed a steeper correlation with anti-HCMV antibody levels (regression analysis slopes were 1.558 vs. 0.258, respectively, left panel). No other FcεRIγ^−^ or FcεRIγ^+^ subset showed a positive correlation with anti-HCMV antibody titre (data not shown). These results indicate that the antiviral antibody response represents a host-dependent factor affecting the size of FcεRIγ^−^ memory NK cell population through the selective expansion of its NKG2C^+^CD57^+^ subset in healthy HCMV^+^ subjects. Although FcεRIγ-expressing NKG2C^+^CD57^+^ shows a moderately higher abundance in individuals with higher anti-HCMV Ab titre, no direct relationship with Ab concentration occurred at the individual level (Figure 2D, right panel), thus implying that the absence of the FcεRIγ chain confers to mature NKG2C^+^CD57^+^ NK cells a higher sensitivity to antiviral antibody levels in vivo, in the context of HCMV chronic infection.

### 3.2. Heterogeneous Sensitivity of Memory NK Cell Subsets to CD16 Stimulation

Memory NK cells’ most distinguishing functional trait is represented by increased responsivity to CD16 prototypical activating receptor [5,6,8,24], whose aggregation unleashes effector functions (cytotoxicity and cytokine production) and promotes proliferation [19,20,21]. 

**Figure 3:**
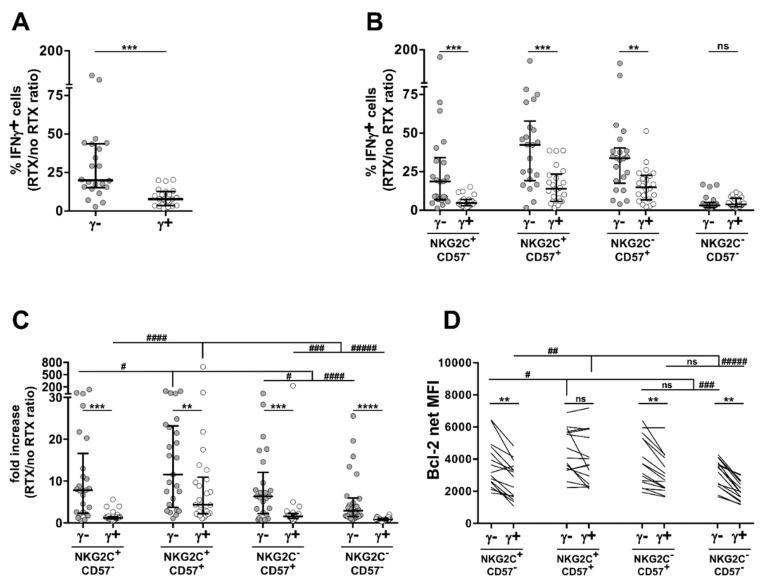
FcεRIγ^−^NKG2C^+^CD57^+^ subset enhanced functional responses to IgG-opsonized targets. (**A,B**) Percentage of IFNγ^−^producing cells upon short-term stimulation with Raji target cells, opsonized or not with rituximab anti-CD20 mAb (RTX), was evaluated in freshly isolated peripheral blood FcεRIγ^−^ (grey) and FcεRIγ^+^ (empty) CD56^dim^ NK cells from HCMV^+^ individuals (**A**), or after stratification according to NKG2C and CD57 expression (**B**). Data are expressed as the fold increase of IFNγ^−^producing cells in the presence vs. absence of rituximab (RTX/no RTX). Data are representative of 22 independent donors. Bars represent median with interquartile range. *p* values of pairwise comparisons were calculated by Wilcoxon non-parametric test. (**) = 0.001, (***) < 0.001. (**C**) Proliferation index of FcεRIγ^−^ (grey) and FcεRIγ^+^ (empty) CD16^+^CD56^dim^ NK cell subsets, evaluated as the ratio between cell number fold increase in the presence vs. absence of rituximab (RTX/no RTX). Data are representative of 28 independent donors. (**D**) Bcl-2 content of FcεRIγ^−^ and FcεRIγ^+^ CD56^dim^ NK cell subsets. Data are expressed as net mean fluorescence intensity (MFI). Data are representative of 13 HCMV^+^ healthy donors. (**C**,**D**) Bars represent median with interquartile range. *p* values of pairwise comparisons were calculated by Wilcoxon and Friedman non-parametric tests, as appropriate. For the sake of clarity, only comparisons with NKG2C^+^CD57^+^ subsets are reported, after Bonferroni correction. (*) symbol indicates statistical comparison with corresponding FcεRIγ^+^ subset (FcεRIγ^−^ vs. FcεRIγ^+^): (**) < 0.005, (***) < 0.00005, (****) < 0.00001; (#) symbol indicates statistical comparison of NKG2C^+^CD57^+^ cells with the other subsets belonging to either FcεRIγ^−^ or FcεRIγ^+^ pools: (#) < 0.05, (##) < 0.005, (###) < 0.0001, (####) < 0.000005, (#####) < 1 × 10^−7^.

Here, we show that FcεRIγ^−^ subsets expressing either CD57 and/or NKG2C displayed a significatively heightened capability to produce IFNγ in response to CD16 stimulation, as compared with their respective FcεRIγ^+^ counterparts (Figure 3B), in accord with the behaviour of the entire FcεRIγ^−^ memory population (Figure 3A). Of note, FcεRIγ^−^NKG2C^+^CD57^+^ cells tendentially showed the highest responsivity among the different FcεRIγ^−^ subsets. 

All FcεRIγ^−^ subsets showed a markedly higher in vitro CD16-driven proliferation index, evaluated as the ratio between the fold increase of proliferation in the presence of opsonized target cells over that attained with not opsonized ones; notably, NKG2C^+^CD57^+^ cell subsets displayed the highest sensitivity to CD16-dependent in vitro expansion within FcεRIγ^−^ and FcεRIγ^+^ NK cell pools (Figure 3C).

Antibody-induced expansion reflects a balance between CD16-induced proliferation and apoptosis [21,22,23]. FcεRIγ^−^ and/or NKG2C^+^ NK cells have been shown to express higher levels of the Bcl-2 anti-apoptotic molecule [8,12]. Here, we show that, with the exception of the NKG2C^+^CD57^+^ subset, all FcεRIγ^−^ subsets display a higher expression of Bcl-2, as compared to their respective FcεRIγ^+^ counterparts (Figure 3D). Of note, FcεRIγ^−^ and FcεRIγ^+^ NKG2C^+^CD57^+^ cells displayed comparable Bcl-2 content that were tendentially highest. 

Collectively taken, these data evidence the higher capability of NKG2C^+^CD57^+^ cells to produce cytokines and proliferate in response to CD16 stimulation; they suggest that higher Bcl-2 levels, by protecting against CD16-dependent pro-apoptotic pathways, may contribute to their CD16-dependent expansion capability. At the same time, our results confirm that the absence of the FcεRIγ chain independently confers higher responsiveness to CD16 engagement and highlight its contribution in explaining the advantage of the FcεRIγ^−^NKG2C^+^CD57^+^ subset over its FcεRIγ^+^ homologue.

### 3.3. In Vivo and In Vitro Exposure to Auto-Antibody-Opsonized Platelets from ITP Patients Impacts Memory NK Cell Subset Profile

The above results indicate that the interaction with antibody-coated cells is an effective driving force that sustains FcεRIγ^−^NKG2C^+^CD57^+^ and, more limitedly, FcεRIγ^+^NKG2C^+^CD57^+^ NK cell expansion in vivo and in vitro. 

To evaluate whether in vivo chronic exposure to opsonizing Ab may affect the profile of memory NK cell subsets in a pathological context, we analysed a cohort of patients affected by immune thrombocytopenia (ITP), a model disease characterized by the presence of anti-platelet and anti-megakaryocyte opsonizing auto-antibodies [36].

We selected HCMV^+^ patients and controls endowed with a sizeable FcεRIγ^−^ pool (>5% of mature CD56^dim^ NK cells), as the abundance of this population positively correlated with the representativity of both FcεRIγ^−^NKG2C^+^CD57^+^ and FcεRIγ^+^NKG2C^+^CD57^+^ cells (Figure 1D). Relevantly, the relative abundance of FcεRIγ^−^ and FcεRIγ^+^ subsets was comparable to that observed in the total cohort of HCMV-seropositive individuals (data not shown).

**Figure 4:**
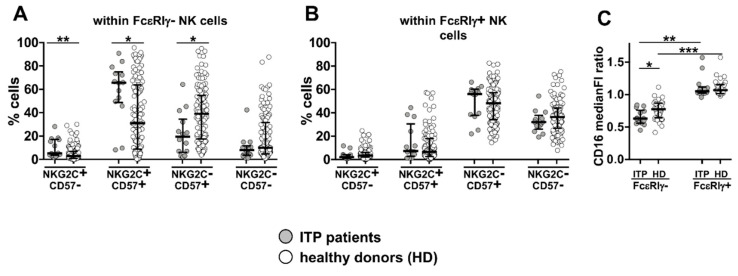
Altered memory NK subset profile in ITP patients. Percentage of FcεRIγ^−^ (**A**) and FcεRIγ^+^ (**B**) CD16^+^CD56^dim^ NK cell subsets in a cohort of 14 ITP patients (grey) and 121 HCMV^+^ healthy individuals (empty), both under 65 years of age, and with a sizeable FcεRIγ^−^ memory pool (>5% of total NK cells). Bars represent median with interquartile range. *p* values of pairwise comparisons were calculated by Mann–Whitney non-parametric test and reported when significant (<0.05): (*) *p* = 0.01, (**) *p* < 0.005. (**C**) CD16 surface levels were evaluated in 14 ITP patients (grey) and 31 age- and sex-matched HCMV^+^ healthy individuals (empty) and expressed as median fluorescence intensity (medianFI) ratio of FcεRIγ^−^ or FcεRIγ^+^ populations vs. medianFI of CD56^dim^ NK cells. Bars represent median with interquartile range. *p* values of pairwise comparisons were calculated by Mann–Whitney and Wilcoxon non-parametric tests, as appropriate, and reported when significant (<0.05): (*) *p* < 0.05, (**) *p* < 0.001, (***) *p* < 0.0001.

Notably, the FcεRIγ^−^NKG2C^+^CD57^+^ subset was significatively expanded in ITP patients, while the relative abundance of its FcεRIγ^+^ counterpart was comparable to that observed in healthy HCMV^+^ individuals (Figure 4A,B, respectively); the more immature FcεRIγ^−^NKG2C^+^CD57^−^ subset was also increased in ITP patients, although to a lower degree. 

In line with previous reports, FcεRIγ^−^ cells were characterized by a lower CD16 expression than FcεRIγ^+^ NK cells in both ITP patients and controls (Figure 4C) [7,24]. Relevantly, the observation that CD16 receptor was further significatively downregulated in FcεRIγ^−^ NK cells of ITP patients is suggestive of an efficient chronic contact with antibody-opsonized cells in vivo (Figure 4C).

**Figure 5:**
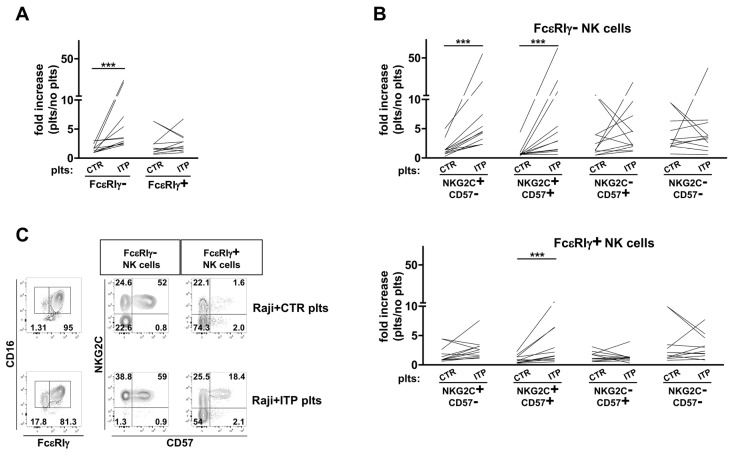
FcεRIγ^−^NKG2C^+^CD57^+^ subset enhanced in vitro expansion driven by ITP patient-derived platelets. Expansion index, expressed as the cell number fold increase in the presence of healthy donor’s (CTR) or ITP patient’s (ITP) platelets (plts) over that attained in the presence of Raji cells alone, of FcεRIγ^−^ and FcεRIγ^+^ NK cells (**A**), or after stratification according to NKG2C and CD57 expression in FcεRIγ^−^ (**B**) (upper panel) and FcεRIγ^+^ (**B**) (lower panel) NK cell pools. (**C**) Plots of a representative HCMV^+^ donor cultured with either CTR or ITP platelets. Numbers represent percentage of gated cells. Data are representative of 12 independent experiments. *p* values of pairwise comparisons were calculated by Wilcoxon non-parametric test and reported when significant (<0.05): (***) *p* < 0.005.

As further evidence that chronic exposure to autoAb-opsonized platelets impacts the profile of memory NK cells, the co-culture with ITP-derived, but not healthy donor-derived, platelets more vigorously induced the expansion of FcεRIγ^−^ NK cells from healthy HCMV^+^ donors, while it did not appreciably affect the proliferation of FcεRIγ^+^ counterpart (Figure 5A). NKG2C^+^CD57^+^ cell proliferation was distinctly dependent on ITP platelet presence in both FcεRIγ^−^ and FcεRIγ^+^ pools; of note, and limitedly to the FcεRIγ^−^ component, the more immature NKG2C^+^CD57^−^ subset showed a more marked expansion in the presence of ITP platelets (Figure 5B upper and lower panel, respectively). 

These data indicate for the first time that CD16-mediated stimulation by opsonizing auto-antibodies led to an amplified in vitro expansion of the FcεRIγ^−^CD16^+^ memory NK cell pool, due to a selective effect on both more (CD57^+^) and less (CD57^−^) differentiated NKG2C^+^ subsets, thus matching the in vivo altered composition of the FcεRIγ^−^ pool in ITP patients (Figure 4A). Differently, only NKG2C and CD57 co-expressing cells displayed a modestly heightened proliferation in response to ITP platelets within the FcεRIγ^+^ pool.

As a whole, our data strongly support the hypothesis that the persistent contact with IgG-opsonized cells may importantly contribute to the shaping, amplification and persistence of the heterogeneous memory NK cell pool in HCMV^+^ individuals in vivo, in either physiological conditions and in a pathological autoimmune setting; they identify the absence of the FcεRIγ chain as a relevant independent factor that amplifies the responsiveness to CD16-dependent activating signals of NKG2C^+^ mature memory NK cells.

## 4. Discussion

Here, we give new insights on the role of opsonizing antibodies in shaping memory NK cell pool composition, establishment and regulation in vivo and in vitro. Our in vivo analysis of memory NK cell distribution encompassed a cohort of HCMV-seropositive healthy individuals and patients affected by ITP, as a model of antibody-mediated autoimmune disease. Our in vitro results directly document the heterogeneous sensitivity of memory NK cell subsets to CD16-initiated signals.

The absence of the FcεRIγ chain, along with the positivity for NKG2C and CD57 receptors [2,5,6,14,15], are phenotypic markers of the memory NK cell pool in humans, although they do not univocally identify the same population [12]. Here, we identify the absence of the FcεRIγ chain as an independent factor that qualifies, within the heterogeneous memory NKG2C^+^CD57^+^ NK phenotype, the subset endowed with marked hyperresponsivity to CD16-dependent stimulation in vitro, and whose higher sensitivity to the presence of opsonizing anti-viral or auto-antibodies determines the expansion level and maintenance in vivo.

HCMV seropositivity was associated with a much larger amplification of the FcεRIγ^−^NKG2C^+^CD57^+^ subset rather than of its FcεRIγ^+^ homologue; the relative abundance of this specific subset largely determines the size of the entire FcεRIγ^−^ pool, as indicated by the strong correlation observed in HCMV-seropositive individuals. 

Our data show that anti-HCMV antibody production represents a host-dependent factor that dictates the size of the FcεRIγ^−^NKG2C^+^CD57^+^ subset at the individual level, and a steeper dependence on anti-HCMV antibody levels is evident in subjects possessing a more expanded population. As an expected consequence, individuals producing higher anti-viral antibody levels are characterized by a larger FcεRIγ^−^ memory pool, on average. The impact of anti-HCMV antibody levels on the FcεRIγ^+^NKG2C^+^CD57^+^ counterpart was more limited, and not apparent at the individual level. These observations suggest that this specific FcεRIγ^+^ subset is responsive, to a lesser extent, to the same factors that regulate its FcεRIγ^−^ counterpart. Additionally, such a population may also represent a precursor for the FcεRIγ^−^NKG2C^+^CD57^+^ subset, as supported by previous observation in a donor with acute HCMV infection [12], suggesting that downregulation of the FcεRIγ chain may occur at a later timepoint during the NK maturation pathway. 

The frequency of NKG2C^+^CD57^+^ or FcεRIγ^−^CD57^+^ NK cells was reported to correlate with anti-HCMV neutralizing antibody levels in a cohort of BMT recipients upon HCMV reactivation [25]. Our results evidence that FcεRIγ^−^NKG2C^+^CD57^+^ is uniquely sensitive to anti-HCMV antibodies in a healthy setting and dissect the contribution of the absence of FcεRIγ in this dependence. The FcεRIγ^−^NKG2C^+^CD57^+^ memory subset was further amplified in HCMV-seropositive patients affected by ITP, despite that anti-HCMV IgG levels were comparable between patients and healthy controls (Capuano C. et al., unpublished data), suggesting for the first time the possibility that opsonizing auto-antibodies can also effectively influence the size of this CD16-hyperresponsive memory subset in humans. 

The lack of FcεRIγ that leads to exclusive coupling of CD16 to the TCRζ adaptor [18] has been mechanistically linked to the functional hyperresponsivity to receptor stimulation in vitro, a cardinal feature of memory identity [7,26]; FcεRIγ^−^ memory NK cell in vitro proliferation induced by contact with virus-infected or tumour cells has been previously demonstrated to depend on the presence of opsonizing Abs [5,6,24]. Our in vitro studies confirm that FcεRIγ^−^ memory subsets have an increased capability to produce cytokines and to proliferate in response to mAb-coated B lymphoma cells, as compared to their FcεRIγ^+^ counterparts. At the same time, both NKG2C^+^CD57^+^ subsets display a higher CD16-dependent responsiveness when compared to the other components within FcεRIγ^−^ and FcεRIγ^+^ pools. This observation reveals that NKG2C^+^ terminally differentiated (CD57^+^) cells are endowed with higher sensitivity to CD16 signalling, independently of the expression of the FcεRIγ protein. The mechanisms underlying the amplified responsiveness to CD16 engagement of FcεRIγ^+^ and FcεRIγ^−^ NKG2C-expressing NK cells are unknown and deserve further investigation. Although recognition of the HLA-E/UL-40 CMV peptide has been shown to directly and efficiently trigger in vitro proliferation of NKG2C^+^ cells in a strain-dependent manner [30], the observation that memory NK cells undergo expansion in NKG2C-deficient individuals suggests that other receptors other than, or in addition to, NKG2C may support HCMV-driven expansion [40,41]; in vitro studies have proposed that synergistic interactions between CD16 and CD2-CD58 axes may fulfil this role [40]. The cooperation between NKG2C and CD16 in memory NK cells could involve transcriptional, epigenetic and metabolic reprogramming mechanisms [3,4,40,42]. The interplay of the signalling pathways initiated by recognition of HLA-E/viral peptide complexes on one side, and interaction with antiviral Ab-opsonized infected cells on the other, may modulate effector capability, proliferative/survival potential and expansion of the memory NK cell pool, as well as affect the linear NK cell maturation pathway in HCMV-infected individuals [2,43]. This cooperation could be further potentiated by the absence of FcεRIγ chain. Higher Bcl-2 intracellular content suggests that increased resistance to CD16-induced apoptosis [22,23] may contribute to the amplified expansion capability of FcεRIγ^−^ subsets and of FcεRIγ^+^NKG2C^+^CD57^+^ cells in response to stimulation with IgG-coated cells in vitro, and to the establishment, maintenance, and expansion in vivo in HCMV^+^ healthy individuals. 

We could document that the interaction with ITP patient-derived platelets selectively promotes in vitro expansion of FcεRIγ^−^NKG2C^+^CD57^+/−^ and, more limitedly, FcεRIγ^+^NKG2C^+^CD57^+^ memory subsets. These data lend mechanistic support to the hypothesis that the augmented abundance of the same subsets observed in HCMV^+^ ITP patients may be due, at least in part, to persistent contact with opsonizing auto-antibodies.

Our work shows for the first time that chronic exposure to opsonizing auto-Abs significantly perturbs memory NK cell compartments in HCMV-seropositive ITP patients, indicating a co-adaptation to both persisting conditions. The memory NK cell skewed immunoregulatory profile, characterized by functional and proliferative hyperresponsivity to IgG-opsonized targets [5,6,8,24], coupled to lower sensitivity to proinflammatory signals and NCR-dependent stimulation [7,9], may well translate in a unique capability to crosstalk with the adaptive arm of the autoreactive response [32,33,34,35]. While memory NK cell contribution is thought to be relevant in some contexts, such as heterologous infections, leukaemia, transplantation and vaccination [43,44,45,46,47,48], its role in autoimmune diseases deserves further investigation. 

In conclusion, our study dissected the quantitative and qualitative heterogeneity of the HCMV-driven memory NK cell compartment and characterized a unique subset whose abundance is associated with anti-HCMV Ab levels in healthy humans and is expanded in ITP patients. Such FcεRIγ^−^NKG2C^+^CD57^+^ population may be envisaged as a fully mature, long-lived NK subset specialized to respond via CD16. The exquisite hypersensitivity to opsonizing antibodies of this specialized memory NK cell subset may well be exploited in a translational perspective, in therapeutical contexts that employ therapeutical mAbs in infectious and non-infectious diseases [49].

## Data Availability

The data presented in this study are available on request from the corresponding authors.

## Supplementary Materials

The following supporting information can be downloaded at: https://www.mdpi.com/article/10.3390/biomedicines10030625/s1, Figure S1: Coordinate expansion of NKG2C^+^, CD57^+^, and FcεRIγ^−^ mature CD56^dim^ NK cells in HCMV-seropositive healthy individuals.

## Abbreviations

Antibody-dependent cytotoxicity (ADCC); type III Fc gamma receptor A (FcγRIIIA); human cytomegalovirus (HCMV); immunoreceptor tyrosine-based activating motif (ITAM); immune thrombocytopenia (ITP); natural cytotoxicity receptors (NCR).
