# Supplementary figures and images for: (Auto)Antibody Responses Shape Memory NK Cell Pool Size and Composition
Source: Biomedicines. 2022 Mar 8;10(3):625. doi: 10.3390/biomedicines10030625 (PMC8945707; doi:10.3390/biomedicines10030625)

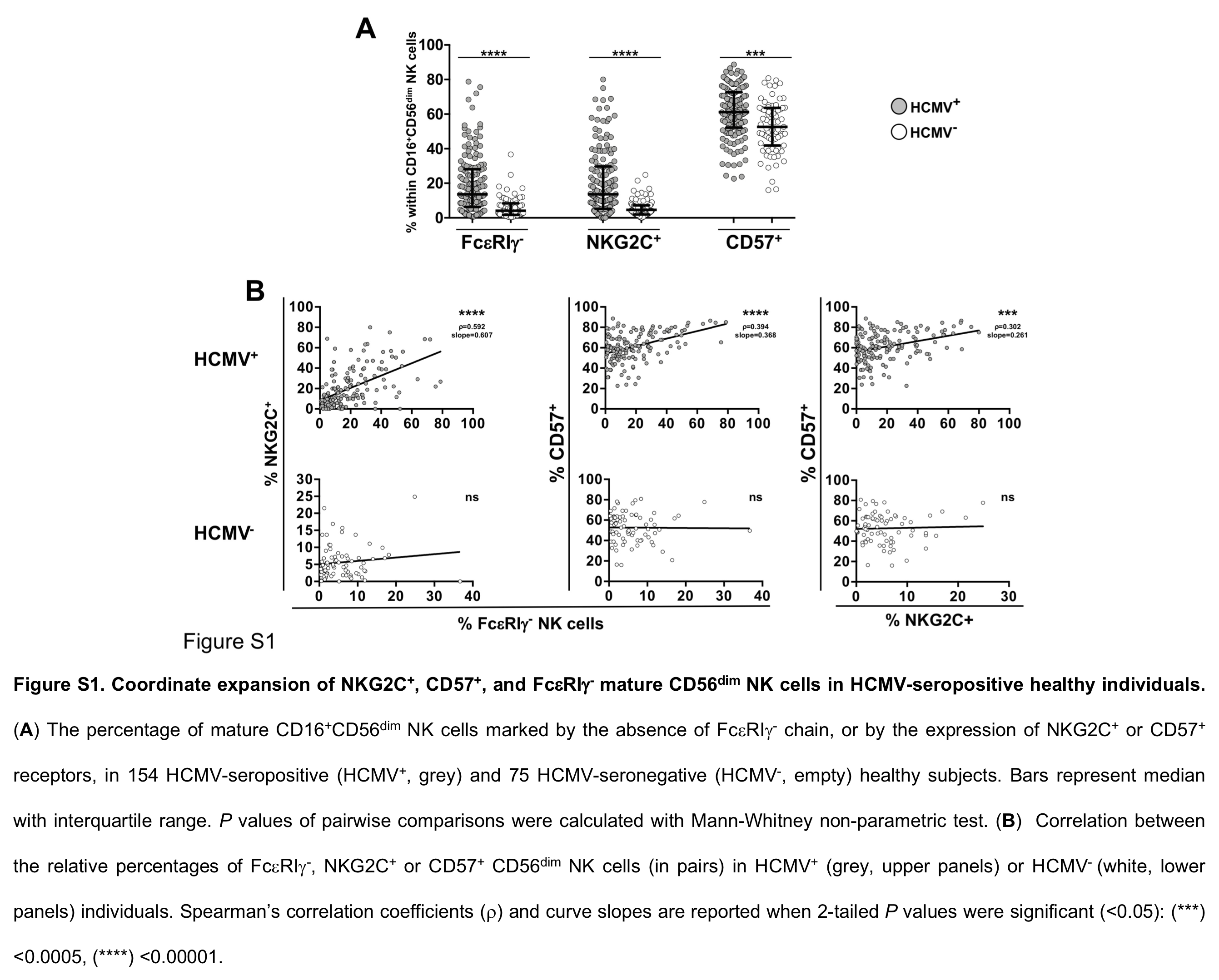

Supplement: Supplementary file 1 [file biomedicines-10-00625-s001.zip › biomedicines-1617726-supplementary-3.8.tiff]
